# Long-term survival of screen-detected synchronous and metachronous bilateral non-palpable breast cancer among Chinese women: a hospital-based study (2003–2017)

**DOI:** 10.1007/s10549-022-06747-5

**Published:** 2022-09-27

**Authors:** Bo Pan, Ying Xu, Yidong Zhou, Ru Yao, Xingtong Zhou, Yali Xu, Xinyu Ren, Mengsu Xiao, Qingli Zhu, Lingyan Kong, Feng Mao, Yan Lin, Xiaohui Zhang, Songjie Shen, Qiang Sun

**Affiliations:** 1 Department of Breast Surgery, Peking Union Medical College Hospital, Chinese Academy of Medical Sciences and Peking Union Medical College, Beijing, 100730 People’s Republic of China; 2 Department of Pathology, Peking Union Medical College Hospital, Chinese Academy of Medical Sciences and Peking Union Medical College, Beijing, 100730 People’s Republic of China; 3 Department of Ultrasound, Peking Union Medical College Hospital, Chinese Academy of Medical Sciences and Peking Union Medical College, Beijing, 100730 People’s Republic of China; 4 Department of Radiology, Peking Union Medical College Hospital, Chinese Academy of Medical Sciences and Peking Union Medical College, Beijing, 100730 People’s Republic of China

**Keywords:** Non-palpable breast cancer (NPBC), Screen-detected, Bilateral, Prognosis, Synchronous, Metachronous

## Abstract

**Purpose:**

Screen-detected unilateral non-palpable breast cancer (NPBC) shows favorable prognosis, whereas bilateral breast cancer (BBC), especially synchronous BBC (SBBC) manifests worse survival than unilateral breast cancer (BC). It remains unclear whether screen-detected bilateral NPBC has compromised survival and requires intensified treatment or favorable prognosis and needs de-escalating therapy.

**Methods:**

From 2003 to 2017, 1,075 consecutive NPBC patients were retrospectively reviewed. There were 988 patients with unilateral NPBC (UniNPBC), and 87 patients with ipsilateral NPBC + any contralateral BC [(N + AnyContra) PBC], including 32 patients with bilateral NPBC (BiNPBC) and 55 patients with ipsilateral NPBC + contralateral palpable cancer [(N + Contra) PBC]. Median follow-up time was 91 (48–227) months. Clinicopathological characteristics were compared between UniNPBC and BBC, whereas relapse-free survival (RFS) and overall survival (OS) among BBC subgroups. RFS and OS factors of BBC were identified.

**Results:**

Compared to UniNPBC, patients with screen-detected bilateral BC had more invasive (85.1%, 74.8%), ER negative (26.4%, 17.1%), PR negative (36.8%, 23.5%), triple-negative (21.6%, 8.5%) BC as well as less breast conserving surgery (17.2%, 32.4%), radiotherapy (13.8%, 32.0%) and endocrine therapy (71.3%, 83.9%). 10 year RFS and OS rates of (N + AnyContra) PBC (72.8%, 81.5%), (N + Contra) PBC (60.6%, 73.9%), and synchronous (N + Contra) PBC (58.1%, 70.1%) were significantly compromised compared to UniNPBC (91.0%, 97.2%). RFS factors of BBC included pN3 (*p* = 0.048), lymphovascular invasion (*p* = 0.008) and existence of contralateral palpable interval BC (*p* = 0.008), while the OS relevant factor was pN3 (*p* = 0.018).

**Conclusion:**

Screen-detected bilateral NPBC including SynBiNPBC and MetaBiNPBC showed good prognosis as UniNPBC so that the therapy of BiNPBC could be de-escalated and optimized according to UniNPBC. Contrarily, screen-detected ipsilateral NPBC with contralateral palpable BC [(N + Contra) PBC] manifested unfavorable survival worse than UniNPBC and synchronous (N + Contra) PBC had the worst survival among all subgroups, implying that these were actually bilateral interval BC and required intensified treatment.

**Supplementary Information:**

The online version contains supplementary material available at 10.1007/s10549-022-06747-5.

## Introduction

Breast cancer (BC) is the commonest malignancy worldwide and the leading cause of cancer death in Chinese women younger than 45 years [1–3]. It is well established that screen-detected BC has favorable biological behavior and prognosis compared to symptomatic interval breast cancer [4–6], which is also true in our previous study on screen-detected non-palpable breast cancer (NPBC) among Chinese asymptomatic women [7]. Meanwhile, we showed in our previous meta-analysis that bilateral breast cancer (BBC), especially synchronous BBC (SBBC) had worse survival compared to unilateral BC (UBC) [8]. However, most studies included in this meta-analysis did not investigate survival of screen-detected BBC, which has increased dramatically over the past 40 years [9]. It remains unclear whether screen-detected bilateral NPBC has compromised prognosis and requires intensified treatment or good survival as unilateral NPBC (UniNPBC) and needs de-escalating therapy. Additionally, it is also a question whether the survival of screen-detected bilateral NPBC would be diversified in view of synchronous or metachronous BBC.

With these questions, we carried out this retrospective study based on long-term follow-up outcomes of a consecutive hospital cohort, to elucidate the specific survival of screen-detected BBC, especially synchronous and metachronous bilateral NPBC and ipsilateral screen-detected NPBC with contralateral palpable interval BC, to provide general and specific data for prognostic evaluation and comparison of screen-detected BBC patient subgroups.

## Patients and methods

### Ethics statement

This retrospective study was approved by the Ethics Committee of the Peking Union Medical College (PUMC) Hospital, Chinese Academy of Medical Sciences.

### Patient cohort, criteria for SBBC vs MBBC diagnosis and follow-up

There were 1075 consecutive female NPBC patients diagnosed in Dept. Breast Surgery, PUMC Hospital from January 2003 to December 2017. There were 988 patients (91.9%) with unilateral screen-detected NPBC (UniNPBC), and 87 patients (8.1%) with BBC of ipsilateral NPBC + any contralateral BC [(N + AnyContra) PBC], including 32 patients with bilateral screen-detected NPBC (BiNPBC) and 55 patients with ipsilateral screen-detected NPBC and contralateral palpable interval cancer [(N + Contra) PBC]. The inclusion criteria were female BC patients 18–90 years old with at least NPBC on one side diagnosed with ultrasound or mammogram guided hook-wire excisional biopsy or core-needle biopsy. Exclusion criteria were male BC patients, patients in pregnancy, bilateral interval/palpable BC and patients < 18 or > 90 years old.

All immunohistochemistry (IHC) staining of ER and PR of BC in PUMC Hospital would be reported with the positivity of nuclear staining percentage. Before 2010, the ER- and PR-positive BC was defined as ER and PR positive staining ≥ 10% by IHC, while patients included from 2011 to 2017 were judged with criteria of ER and PR staining ≥ 1% in IHC as positive according to the guidelines from the American Society of Clinical Oncology (ASCO) and College of American Pathologists (CAP) [10]. When IHC staining of Her2 was (2 +), fluorescence in situ hybridization (FISH) was used to determine the Her2 status. Before the year of 2013, HER2 to centromere enumerator probe (CEP) 17 ratio on FISH ≥ 2.2 was taken as positive, 1.8–2.2 as equivocal and < 1.8 as negative. Since 2014, the FISH criteria was changed to ≥ 2.0 as positive and < 2.0 as negative [11].

Metachronous BBC (MBBC) was diagnosed if the interval between first and second BC diagnosis was > 6 months [8]. The intervals of the 29 MBBC patients were 12–164 (median 61) months. The 32 BiNPBC patients included 20 synchronous bilateral screen-detected NPBC (SynBiNPBC) and 12 metachronous bilateral NPBC (MetaBiNPBC). The 55 (N + Contra) PBC patients included 38 synchronous bilateral BC with ipsilateral NPBC and contralateral palpable BC [Syn (N + Contra) PBC] as well as 17 metachronous BBC [Meta (N + Contra) PBC]. Among the 17 Meta (N + Contra) PBC patients, 9 NPBC were diagnosed before the contralateral palpable BC (ContraPBC), whereas 8 NPBC came after the ContraPBC.

All patients were followed by telephone call, out-patient clinics records of follow-up examinations or by both measures. The follow-up time was 48–227 (median 91) months.

### Comparsion of clinicopathological characteristics and survival

The clinicopathological characteristics were compared between UniNPBC and (N + AnyContra) PBC patients as well as between BiNPBC and (N + Contra) PBC tumors with exclusion of the ContraPBC. The comparisons of Her2 status and subtype were performed among invasive cancers only. The 10 year relapse-free survival (RFS) and overall survival (OS) were compared among UniNPBC, (N + AnyContra) PBC, BiNPBC and subgroups, (N + Contra) PBC and subgroups, all synchronous BBC and all metachronous BBC, as well as among SynBiNPBC, MetaBiNPBC, Syn (N + Contra) PBC, and Meta (N + Contra) PBC.

RFS of synchronous BBC was defined as the interval between diagnoses of index cancer and the first recurrence or metastasis. As for metachronous BBC, the development of contralateral BC would be taken as a relapse event. Thus, RFS of the first cancer was the time interval between its diagnosis and the first RFS event including second (contralateral) cancer, while RFS of the second cancer was the time interval of its diagnosis and the next relapse event. The RFS definition would be further discussed in the Discussion of this article.

### Statistical analysis

The quantitative variables were compared with *t*-test, the categorical variables with chi-square tests, and survival outcomes by the Kaplan–Meier curve method. RFS and OS related prognostic factors of BBC [(N + AnyContra) PBC] were identified, respectively, by Kaplan–Meier univariate analyses and Cox multivariate analyses. The significance threshold was set at *p* < 0.05. R (v4.1.3) software was used for all of the statistical analyses.

## Results

### Comparison of clinicopathological characteristics between screen-detected unilateral and bilateral BC

Compared to UniNPBC patients, screen-detected bilateral BC patients [(N + AnyContra) PBC] had more invasive BC (85.1% vs. 74.8%, *p* = 0.033), ER negative BC (26.4% vs. 17.1%, *p* = 0.032), PR negative BC (36.8% vs. 23.5%, *p* = 0.007), triple-negative BC (TNBC, 18.4% vs. 6.4%, *p* = 0.008), less breast conserving surgery (BCS, 17.2% vs. 32.4%, *p* = 0.013), less radiotherapy (13.8% vs. 32.0%, *p* < 0.001), and less endocrine therapy (71.3% vs. 83.9%, *p* = 0.002) (Table 1). There were no significant differences in age at first BC diagnosis, TNM stage, histological grade, multi-focality, lymphovascular invasion (LVI), Ki67 index, chemotherapy, and Her2-targeted therapy (Table 1).

**Table 1:** Comparison of clinicopathological characteristics of patients with screen-detected unilateral and bilateral breast cancer among Chinese women

Clinicopathological characteristics	UniNPBC *N* = 988 (%)	(N + Anycontra) PBC *N* = 87 (%)	*P* value
Age (1st cancer)	50.6 ± 11.6	53.2 ± 13.5	0.091
Age group (1st cancer)			0.134
< 40	150 (15.2)	13 (14.9)	
40 ~ 49	367 (37.1)	22 (25.3)	
50 ~ 59	255 (25.8)	29 (33.3)	
≥ 60	216 (21.9)	23 (26.4)	
Tumor histology			**0.033**
DCIS	249 (25.2)	13 (14.9)	
Invasive	739 (74.8)	74 (85.1)	
pT			0.101
Tis	249 (25.2)	13 (14.9)	
T1	675 (68.3)	68 (78.2)	
T2	64 (6.5)	6 (6.9)	
Lymph node status			0.707
Negative	825 (83.5)	74 (85.1)	
Positive	163 (16.5)	13 (14.9)	
pN			0.884^#^
N0	825 (83.5)	74 (85.1)	
N1	115 (11.6)	9 (10.3)	
N2	22 (2.2)	1 (1.1)	
N3	26 (2.6)	3 (3.4)	
TNM stage			0.101^#^
0	249 (25.2)	13 (14.9)	
I	543 (55.0)	59 (67.8)	
II	149 (15.1)	11 (12.6)	
III	47 (4.8)	4 (4.6)	
Histological grade			0.356*
G1	218 (22.1)	16 (18.4)	
G2	493 (49.9)	50 (57.5)	
G3	224 (22.7)	16 (18.4)	
Unknown	53 (5.4)	5 (5.7)	
Focality			0.119
Unifocal	845 (85.5)	69 (79.3)	
Multifocal	143 (14.5)	18 (20.7)	
LVI			0.299
No	944 (95.5)	81 (93.1)	
Yes	44 (4.5)	6 (6.9)	
ER			0.032*
Negative	169 (17.1)	23 (26.4)	
Positive	811 (82.1)	64 (73.6)	
Unknown	8 (0.8)	/	
PR			0.007*
Negative	232 (23.5)	32 (36.8)	
Positive	749 (75.8)	55 (63.2)	
Unknown	7 (0.7)	/	
Her2 (invasive BC)			0.702*
Negative	616 (83.4)	63 (85.1)	
Positive	112 (15.2)	10 (13.5)	
Unknown	11 (1.5)	1 (1.4)	
Ki67			0.613*
< 14%	486 (49.2)	41 (47.1)	
≥ 14%	476 (48.2)	45 (51.7)	
Unknown	26 (2.6)	1 (1.1)	
Subtype (invasive BC)			0.008*^#^
Luminal A	271 (36.7)	20 (27.0)	
Luminal B	330 (44.7)	31 (41.9)	
Her2	44 (6.0)	4 (5.4)	
TNBC	63 (8.5)	16 (21.6)	
Unknown	31 (4.2)	3 (4.1)	
Surgery			**0.013**^#^
Mastectomy	668 (67.6)	69 (79.3)	
Breast conserving	320 (32.4)	15 (17.2)	
Mastectomy + BCS^$^	/	3 (3.4)	
Chemotherapy			0.484
No	650 (65.8)	54 (62.1)	
Yes	338 (34.2)	33 (37.9)	
Radiotherapy			< 0.001*
No	670 (67.8)	75 (86.2)	
Yes	316 (32.0)	12 (13.8)	
Unknown	2 (0.2)	/	
Her2 targeted therapy			0.443*
No	898 (90.9)	77 (88.5)	
Yes	79 (8.0)	9 (10.3)	
Unknown	11 (1.1)	1 (1.1)	
Endocrine therapy			0.002*
No	153 (15.5)	25 (28.7)	
Yes	829 (83.9)	62 (71.3)	
Unknown	6 (0.6)	/	

*BC* breast cancer, *NPBC* non-palpable breast cancer, *UniNPBC* unilateral non-palpable breast cancer, *(N* + *AnyContra) PBC* ipsilateral NPBC with any contralateral breast cancer, *DCIS* ductal carcinoma in situ, *LVI* lymphovascular invasion, *ER* estrogen receptor, *PR* progesterone receptor, *TNBC* triple-negative breast cancer, *BCS* breast-conserving surgery

*The comparison was performed without the unknown cases, otherwise there would have been significant difference caused by the unknown cases

^#^The comparison was performed by Fisher’s test

^$^Bilateral breast cancer patients who underwent mastectomy on one side and breast conserving surgery on the other side

Bold *P*-value suggested significance in comparison

**Fig. 1:**
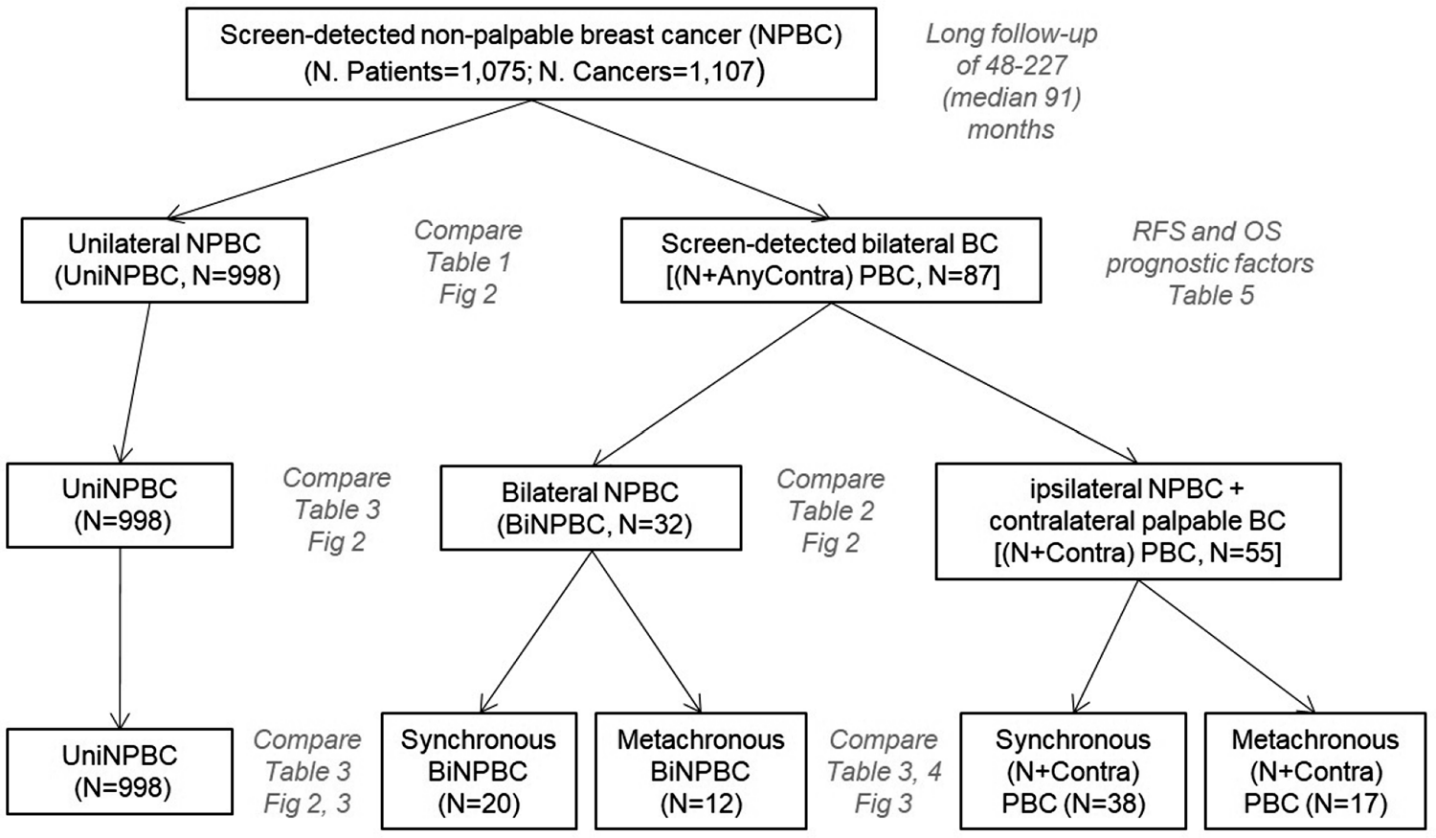
Flowchart of the study design with case number of each subgroup of screen-detected unilateral and bilateral NPBC. The table and figure illustrating each comparison results was italicized and in gray

In view of the tumors, when the contralateral palpable BCs were excluded, the 64 screen-detected NPBC of the 32 BiNPBC patients were similar compared to the 55 screen-detected NPBC of 55 (N + AnyContra) PBC patients in terms of age at diagnosis, percentage of invasive BC, TNM stage, histological grade, multi-focality, ER, PR, Her2, Ki67 index, subtype, surgery, radiotherapy, Her2-targeted therapy, and endocrine therapy (Table 2). (N + AnyContra) PBC received more chemotherapy than BiNPBC (43.6% vs. 23.4%, *p* = 0.019) and showed an insignificant trend of more LVI (9.1% vs. 1.7%, synchronous 15.2% vs. 2.6%, *p* = 0.094) (Table 2, Fig. 1).

**Table 2:** Clinicopathological characteristics of screen-detected synchronous and metachronous bilateral NPBC as well as ipsilateral NPBC with contralateral palpable breast cancer

Clinicopathological characteristics	Screen-detected synchronous and metachronous bilateral NPBCs								*P* value^&^
	BiNPBC (*N* = 32 × 2 = 64)					(*N* + Contra) PBC (*N* = 55)			
	All (*N* = 64)	SynBiNPBC (*N* = 20 × 2 = 40)		MetaBiNPBC (*N* = 12 × 2 = 24)		All (*N* = 55)	Syn(*N* + Contra) PBC (*N* = 38)	Meta(*N* + Contra) PBC (*N* = 17)	
		Index	Contralateral	Index	Contralateral				
Age									
(M ± SD)	55.4 ± 12.3	57.8 ± 14.2	57.8 ± 14.2	48.9 ± 6.8	53.7 ± 7.2	52.4 ± 14.1	54.3 ± 15.3	48.2 ± 10.1	0.234
< 40	5	2	2	1	0	10	7	3	0.275
40 ~ 49	17	4	4	5	4	13	8	5	
50 ~ 59	20	5	5	5	5	19	11	8	
≥ 60	22	9	9	1	3	13	12	1	
Tumor histology									0.400
DCIS	17	2	10	3	2	11	7	4	
Invasive	47	18	10	9	10	44	31	13	
pT									0.714^#^
Tis	17	2	10	3	2	11	7	4	
T1	44	17	10	8	9	41	29	12	
T2	3	1	0	1	1	3	2	1	
LN status									0.554
Negative	57	18	19	11	9	47	33	14	
Positive	7	2	1	1	3	8	5	3	
pN									0.162^#^
N0	57	18	19	11	9	47	33	14	
N1	7	2	1	1	3	4	2	2	
N2	0	0	0	0	0	1	0	1	
N3	0	0	0	0	0	3	3	0	
TNM stage									0.131^#^
CIS	18	2	10	3	2	11	7	4	
I	38	15	9	7	8	35	25	10	
II	8	3	1	2	2	5	3	2	
III	0	0	0	0	0	4	3	1	
Histological grade									0.760*
G1	11	1	6	2	2	13	13	0	
G2	35	13	8	6	8	29	20	9	
G3	12	4	4	2	2	11	5	6	
Unknown	6	2	2	2	0	2	0	2	
Focality									0.863
Unifocal	52	16	19	9	8	44	29	15	
Multifocal	12	4	1	3	4	11	9	2	
LVI									0.094^#^
No	63	19	20	12	12	50	33	17	
Yes	1	1	0	0	0	5	5	0	
ER									0.971*
Negative	17	4	3	4	6	15	9	6	
Positive	46	16	16	8	6	40	29	11	
Unknown	1	0	1	0	0	0	0	0	
PR									0.921*
Negative	22	6	3	5	8	20	13	7	
Positive	40	14	15	7	4	35	25	10	
Unknown	2	0	2	0	0	0	0	0	
Her2 (invasive BC)									0.631*
Negative	42	15	10	8	9	37	26	11	
Positive	5	3	0	1	1	6	5	1	
Unknown	0	0	0	0	0	1	0	1	
Ki67									0.768^#*^
< 14%	31	7	12	7	5	29	20	9	
≥ 14%	31	13	7	4	7	26	18	8	
Unknown	2	0	1	1	0	0	0	0	
Subtype (invasive BC)									0.757^#^*
Luminal A	14	3	4	3	4	13	11	2	
Luminal B	22	12	5	2	3	16	11	5	
Her2	2	1	0	1	0	3	3	0	
TNBC	8	2	1	1	4	10	5	5	
Unknown	3	0	1	2	0	2	1	1	
Surgery									0.338
BCS	15	5	5	2	3	9	7	2	
Mx	49	15	15	10	9	46	31	15	
Chemotherapy									**0.019**
No	49	15	17	9	8	31	23	8	
Yes	15	5	3	3	4	24	15	9	
Radiotherapy									0.970
No	56	18	18	10	10	48	33	15	
Yes	8	2	2	2	2	7	5	2	
Her2 targeted therapy									0.756*
No	58	18	19	11	10	48	33	15	
Yes	6	2	1	1	2	6	5	1	
Unknown	0	0	0	0	0	1	0	1	
Endocrine therapy									0.885
No	19	4	6	3	6	17	10	7	
Yes	45	16	14	9	6	38	28	10	

*NPBC* non-palpable breast cancer, *UniNPBC* unilateral non-palpable breast cancer, *(N* + *Contra) PBC* ipsilateral NPBC with contralateral palpable breast cancer, *SynBiNPBC* synchronous bilateral NPBC, *MetaBiNPBC* metachronous bilateral NPBC, *Syn(N* + *Contra)PBC* synchronous ipsilateral NPBC with contralateral palpable breast cancer, *Meta(N* + *Contra)PBC* metachronous ipsilateral NPBC with contralateral palpable breast cancer, *DCIS* ductal carcinoma in situ, *LVI* lymphovascular invasion, *ER* estrogen receptor, *PR* progesterone receptor, *TNBC* triple-negative breast cancer, *Mx* mastectomy, *BCS* breast-conserving surgery

*The comparison was performed without the unknown cases, otherwise there would have been significant difference caused by the unknown cases

^#^The comparison was performed by Fisher’stest

^&^The comparison was performed in all BiNPBC and all (N + Contra) PBC

### Survival comparison among screen-detected unilateral, bilateral, synchronous and metachronous subgroups of NPBC

**Fig. 2:**
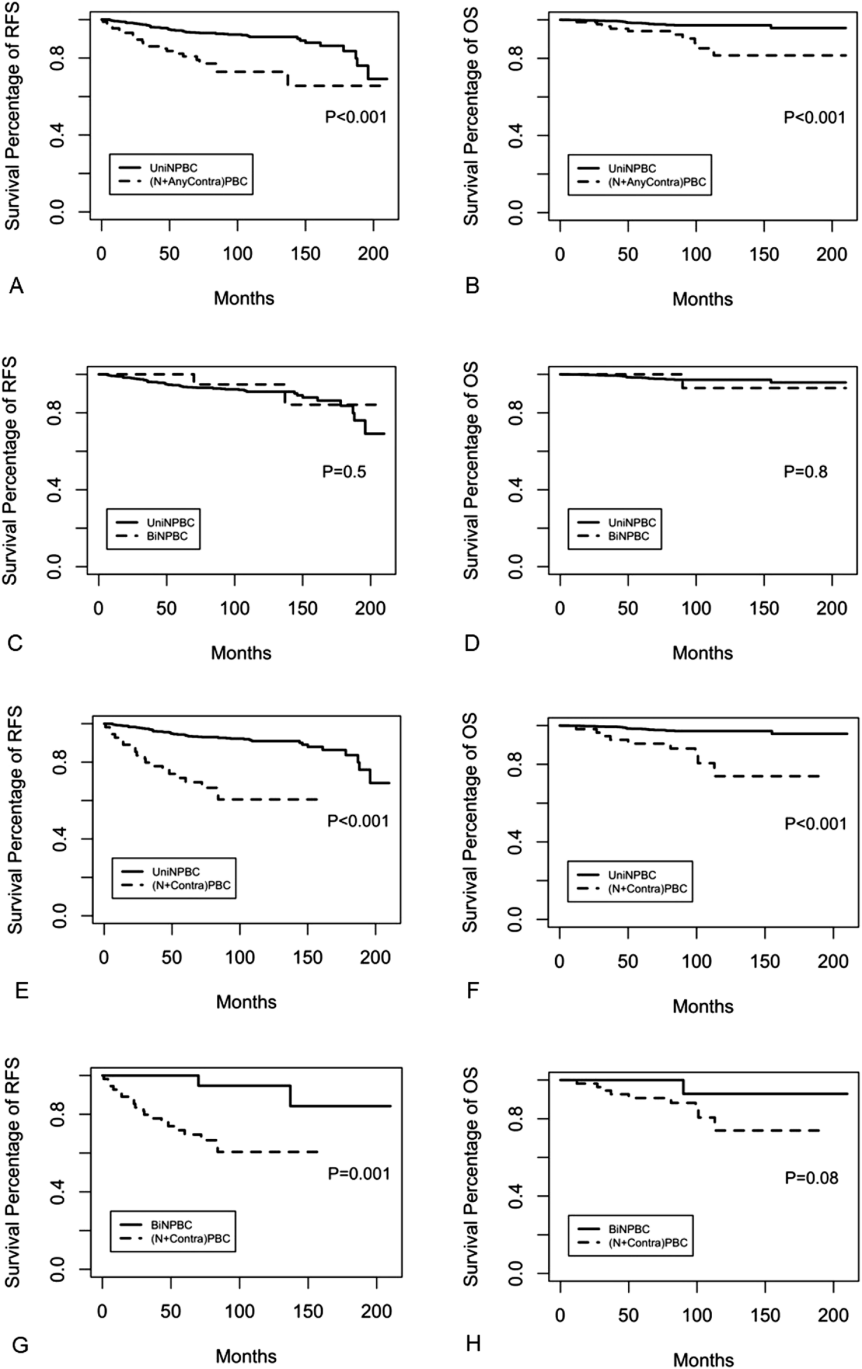
Comparison of RFS and OS between UniNPBC vs (N + AnyContra) PBC (**A**, **B**), UniNPBC vs BiNPBC (**C**, **D**), UniNPBC versus (N + Contra) PBC (**E**, **F**) and BiNPBC vs (N + Contra) PBC (**G**, **H**). BiNPBC showed similar good prognosis as UniNPBC (**C**, **D**), whereas survival of (N + AnyContra) PBC and (N + Contra) PBC were significantly worsened than UniNPBC (**E**–**H**)

**Fig. 3:**
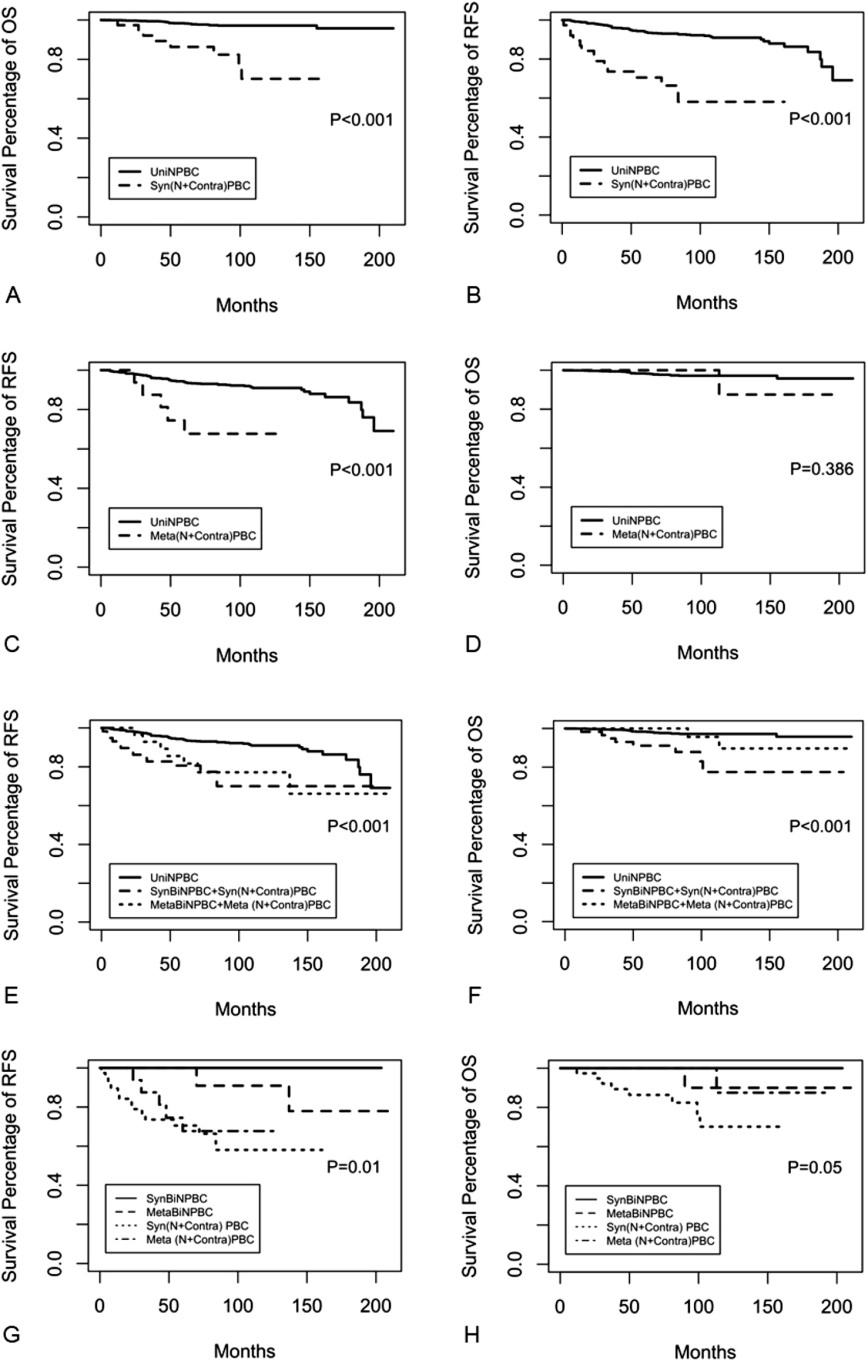
Comparison of RFS and OS between UniNPBC vs synchronous (N + Contra) PBC (**A**, **B**), vs metachronous (N + Contra) PBC (C, D), vs all synchronous and metachronous BBC (**E**, **F**) and comparison among subgroups of BiNPBC and (N + Contra) PBC (**G**, **H**). Synchronous (N + Contra) showed the worst survival (**C**, **D**), while SynBiNPBC manifested the best prognosis (**G**, **H**)

In terms of 10-year RFS and OS rates, BiNPBC (94.7% and 92.9%), synchronous (SynBiNPBC, 100% and 100%), and metachronous BiNPBC (MetaBiNPBC, 90.9% and 90.0%) showed similar good prognosis as UniNPBC (91.0% and 97.2%, Table 3, Fig. 2). However, survival of (N + AnyContra) PBC (72.8% and 81.5%), (N + Contra) PBC (60.6% and 73.9%), synchronous (N + Contra) PBC (58.1 and 70.1%) and synchronous BBC [SynBiNPBC + Syn (N + Contra) PBC] (70.0% and 77.4%) were all significantly compromised when compared to those of UniNPBC (all the *p* < 0.001, Table 3, Figs. 2, 3). The 10-year RFS of BiNPBC was higher than 10-year OS rate due to RFS event happened later than 120 months. The 15-year RFS rate of BiNPBC was 84.2% (HR 65.3–100.0) and the 15-year OS rate 92.9% (HR 80.3–100.0). The 10-year RFS rate of metachronous (N + Contra) PBC (67.7%) and metachronous BBC [MetaBiNPBC + Meta (N + Contra) PBC, 77.1%] were worsened than that of UniNPBC (91.0%, both *p* < 0.001, Table 3), whereas there was no significant difference among 10-year OS of these three subgroups (87.5% and 89.7% vs. 97.2%, Table 3, Figs. 2, 3).

**Table 3:** Comparison of long-term survival of screen-detected unilateral NPBC, bilateral NPBC and its synchronous and metachronous subgroups, ipsilateral NPBC with contralateral palpable breast cancer and its subgroups as well as all synchronous and metachronous screen-detected BC

Screen-detected NPBC subgroups	*N*	10 year RFS (%) (95%CI)	HR* of RFS	*P* value of RFS	10 year OS (%) (95%CI)	HR* of OS	*P* value of OS
UniNPBC	988	91.0 (88.8–93.2)	Ref	Ref	97.2 (96.0–98.3)	Ref	Ref
(*N* + Anycontra) PBC	87	72.8 (62.9–84.4)	3.79 (2.27–6.33)	< 0.001	81.5 (70.7–94.0)	6.55 (2.93–14.67)	< 0.001
BiNPBC	32	94.7 (85.2–100.0) ^#^	0.53 (0.12–2.37)	0.414	92.9 (80.3–100.0)^#^	1.29 (0.14–11.98)	0.823
SynBiNPBC	20	100	–	0.994	100	–	0.998
MetaBiNPBC	12	90.9(75.4–100) ^#^	1.10(0.91–4.92)	0.901	90.0 (73.2–100)^#^	2.70 (0.30–24.23)	0.376
(*N* + Contra) PBC	55	60.6 (47.7–76.9)	9.73 (6.07–15.60)	< 0.001	73.9 (58.4–93.4)	9.89 (4.23–23.11)	< 0.001
Syn (*N* + Contra) PBC	38	58.1(42.9–78.6)	8.12(4.44–14.84)	< 0.001	70.1 (53.3–92.3)	15.33 (6.12–38.40)	< 0.001
Meta (*N* + Contra)PBC	17	67.7(47.9–95.7)	5.33(2.07–13.7)	< 0.001	87.5 (67.3–100)	2.60 (0.32–20.94)	0.368
SynBiNPBC + Syn (*N* + Contra) PBC	58	70.0 (57.0–86.0)	4.57 (2.58–8.28)	< 0.001	77.4 (63.0–95.2)	9.74 (4.35–21.79)	< 0.001
MetaBiNPBC + Meta (*N* + Contra) PBC	29	77.1 (62.5–95.2)	4.52 (2.49–8.21)	< 0.001	89.7 (76.9–100.0)	2.60 (0.62–10.93)	0.191

*NPBC* non-palpable breast cancer, *UniNPBC* unilateral non-palpable breast cancer, *(N* + *Contra) PBC* ipsilateral NPBC with contralateral palpable breast cancer, *SynBiNPBC* synchronous bilateral NPBC, *MetaBiNPBC* metachronous bilateral NPBC, *Syn(N* + *Contra)PBC* synchronous ipsilateral NPBC with contralateral palpable breast cancer, *Meta(N* + *Contra)PBC* metachronous ipsilateral NPBC with contralateral palpable breast cancer, *RFS* relapse-free survival, *OS* overall survival

*HR hazard ratio adjusted for age, TNM stage tumor grade, subtype, surgery, chemotherapy, radiotherapy, endocrine therapy and target therapy

^#^The 10 year RFS was higher than 10 year OS rate due to RFS event happened later than 120 months. The 15 year RFS rate of BiNPBC was 84.2% (65.3–100.0) and the 15 year OS rate 92.9% (80.3–100.0), whereas the 15 year RFS rate of MetaBiNPBC was 77.9% (54.6–100.0) and the 15 year OS rate 90.0% (73.2–100)

As for subgroups of screen-detected BBC in terms of synchronous and metachronous BBC, SynBiNPBC showed the best 10-year RFS and OS rate of both 100%. Compared to SynBiNPBC, these prognostic counterparts were significantly worsened of MetaBiNPBC (90.9% and 90.0%), Syn (N + Contra) PBC (58.1% and 70.1%), and Meta (N + Contra) PBC (67.7% and 87.5%) (Table 4, Fig. 3). The 10-year RFS of MetaBiNPBC was higher than 10-year OS rate due to RFS event happened later than 120 months. The 15-year RFS rate of MetaBiNPBC was 77.9% (54.6–100.0) and the 15-year OS rate 90.0% (73.2–100) (Table 4).

**Table 4:** Comparison of long-term survival of screen-detected synchronous and metachronous bilateral NPBC as well as synchronous and metachronous ipsilateral NPBC with contralateral palpable breast cancer

Bilateral NPBC subgroups	*N*	10 year RFS% (95%CI)	*P* value of RFS	10 year OS% (95%CI)	*P* value of OS
SynBiNPBC	20	100	Ref	100	Ref
MetaBiNPBC	12	90.9(75.4–100) ^#^	< 0.001	90.0(73.2–100) ^#^	< 0.001
Syn (*N* + Contra) PBC	38	58.1(42.9–78.6)	< 0.001	70.1(53.3–92.3)	< 0.001
Meta (*N* + Contra) PBC	17	67.7(47.9–95.7)	< 0.001	87.5(67.3–100)	< 0.001

*NPBC* non-palpable breast cancer, *(N* + *Contra) PBC* ipsilateral NPBC with contralateral palpable breast cancer, *SynBiNPBC* synchronous bilateral NPBC, *MetaBiNPBC* metachronous bilateral NPBC, *Syn(N* + *Contra)PBC* synchronous ipsilateral NPBC with contralateral palpable breast cancer, *Meta(N* + *Contra)PBC* metachronous ipsilateral NPBC with contralateral palpable breast cancer, *RFS* relapse-free survival, *OS* overall survival

*HR hazard ratio was not calculated due to limited number of cases and survival events

^#^The 10 year RFS was higher than 10 year OS rate due to RFS event happened later than 120 months. The 15 year RFS rate of MetaBiNPBC was 77.9% (54.6–100.0) and the 15 year OS rate 90.0% (73.2–100)

### Identification of RFS and OS prognostic factors of all screen-detected BBC

Among all screen-detected BBC, the RFS related prognostic factors included pN3 (*p* = 0.048), lymphovascular invasion (LVI, *p* = 0.008) and the existence of contralateral palpable interval BC (*p* = 0.008), while the OS relevant factor was pN3 (*p* = 0.018) (Table 5).

**Table 5:** Univariate and multivariate Cox analysis of RFS and OS related prognostic factors of screen-detected bilateral breast cancer patients

Variables	RFS			OS		
	Univariate	Multivariate		Univariate	Multivariate	
	*P* value	HR (95% CI)	*P* value	*P* value	HR (95% CI)	*P* value
Age at diagnosis	0.1	–	–	0.1	–	–
Histological type	0.7	–	–	0.5	–	–
pT	0.7	–	–	0.8	–	–
Lymph node status	0.05	–	–	**0.02**	–	–
pN	** < 0.001**			** < 0.001**		
N0		Ref			Ref	
N1		0.99(0.25–4.05)	0.999		1.67(0.19–14.44)	0.640
N2		–	0.998		–	0.998
N3		5.53(1.01–30.11)	**0.048**		27.2 (1.74–423.15)	**0.018**
TNM stage	0.1	–	–	**0.01**		
DCIS		–	–		Ref	
I		–	–		1.55(0.17–13.97)	0.698
II		–	–		2.16(0.12–39.50)	0.603
III		–	–		–	–
Focality	0.5	–	–	1	–	–
LVI	** < 0.001**			**0.03**		
No		Ref			Ref	
Yes		4.68(1.51–14.55)	**0.008**		3.07(0.56–16.92)	0.198
Contralateral palpable interval BC	**0.006**			0.113		
No		Ref			–	–
Yes		8.67(1.73–43.32)	**0.008**		–	–
ER status	0.4	–	–	0.7	–	–
PR status	0.6	–	–	0.8	–	–
HER2 status	0.7	–	–	0.5	–	–
Ki67 index	0.5	–	–	0.8	–	–
Subtype	0.4	–	–	0.4	–	–
Surgery	0.6	–	–	0.05	–	–
Chemotherapy	**0.03**			0.2	–	–
No		Ref				
Yes		2.36(0.81–6.84)	0.116			
Radiotherapy	0.8	–	–	0.3	–	–
Anti-Her2 targeted therapy	0.6	–	–	0.5	–	–
Endocrine therapy	0.6	–	–	0.9	–	–

*BC* breast cancer, *DCIS* ductal carcinoma in situ, *LVI* lymphovascular invasion, *ER* estrogen receptor, *PR* progesterone receptor

Bold *P*-value suggested significance in comparison

*The comparison was performed without the unknown cases, otherwise there would have been significant difference caused by the unknown cases

## Discussion

The growing awareness, prolonged lifetime, advancements in diagnostic imaging, and improvements in detection rate from diversified screening had resulted in increased incidence of BBC and SBBC [2, 3, 12, 13]. Studies on survival of screen-detected BBC are difficult to conduct because there are several issues bringing complexity: (1) the heterogeneity of contralateral BC: for ipsilateral NPBC, the contralateral BC could be screen-detected NPBC, screen-detected symptomatic BC or interval BC; (2) the parameter of diagnostic interval: the contralateral BC could be synchronous or metachronous; (3) the parameter of diagnostic sequence: for ipsilateral NPBC with contralateral palpable or interval BC, would it be different whether the ipsilateral NPBC comes first or after the contralateral BC?

To our knowledge, our study was the first to investigate the long-term prognosis of screen-detected synchronous and metachronous BBC with the concern of contralateral palpable or non-palpable BC among Chinese women. The BC screening in China is quite diversified [3, 7, 12, 14, 15] so the screen-detected symptomatic BC and interval BC were usually difficult to differentiate and both documented as contralateral palpable BC in our study. The long-term follow-up of median 91 months was to ensure accurate evaluation of personalized prognosis and sufficient detection of metachronous BBC. To decipher the complexity of prognosis of screen-detected BBC, we choose interval of 6 months between detection of bilateral cancers as the criteria to differentiate SBBC versus MBBC to minimize the confounding effects on comparison of survival [8]. As for the third issue mentioned above, there were only 9 NPBC diagnosed before contralateral palpable BC, whereas 8 NPBC came after, which was too small case number for survival comparison. Studies suggested that risk of third primary cancers of non‐breast origin among women with BBC would also increase, indicating that BBC might be genetically susceptible to develop cancer [16]. Hence we chose RFS in addition to OS as prognostic endpoint so that the relapse events were breast cancer specific and diseases such as third primary cancers including thyroid, lung, pancreas, or cardiovascular diseases, etc. would be excluded as RFS events.

In our study, the development of contralateral BC in MBBC would also be taken as a relapse event. Otherwise, the survival outcome of the first tumor (ipsilateral BC) of MBBC would be over-estimated. For example, the contralateral BC showed different histology or subtype from ipsilateral BC, and then recurrence arose years later from the contralateral BC, if the development of contralateral BC was not taken as a relapse event, it was also unreasonable to take this recurrence from contralateral BC as a relapse event of the ipsilateral BC, then the RFS of the first tumor (ipsilateral BC) would be largely biased and over-estimated too much.

As for the clinicopathological features, study suggested that index cancers of bilateral screen-detected cancers and bilateral interval cancers show significant differences in tumor size, whereas nodal status, receptor status, and final surgical treatment are comparable [17]. Our study showed that the clinicopathological characteristics of screen-detected NPBC in those (N + Contra) PBC patients were similar to those of BiNPBC (Table 2). Taken together, it implied that screen-detected NPBC, either bilateral or ipsilateral, might be different clinical entities with contralateral palpable interval BC, even in the same patient and regardless of synchrony or metachrony.

There were controversies about whether adjuvant therapy for BBC should base on the higher risk tumor or the index tumor [18] and adjuvant chemotherapy might paradoxically both reduce the risk and worsen the prognosis of MBBC [13]. For screen-detected BBC, this paradox also included escalation or de-escalation of the treatment. Our study showed (N + Contra) PBC received more chemotherapy than BiNPBC due to existence of contralateral palpable BC (Table 2), however (N + Contra) PBC still showed worsened survival than BiNPBC, suggesting that the treatment of (N + Contra) PBC should be escalated. Similar to reports from other studies that majority of BBC patients (69.0–76.2%) would usually choose bilateral mastectomy even with young age [9, 19, 20], (N + AnyContra) PBC patients in our study also received more mastectomy compared to UniNPBC (79.3% vs. 67.6%, Table 1).

Study implied that women were more likely to have small breast cancer that was detected in screening than to have earlier detection of a tumor that was destined to become large [5]. In other words, early BC including NPBC could be detected small because they were good in biological behavior rather than they were good in prognosis because they were detected small. Our study results were highly coincided with this. Bilateral NPBC (BiNPBC) was actually screen-detected low-risk BC with similar prognosis as unilateral NPBC (UniNPBC) (Table 3, Fig. 2). Thus, the therapy of BiNPBC could be tailored according to UniNPBC and should not be intensified as those symptomatic SBBC. On the contrary, if the contralateral BC was palpable [(N + Contra) PBC], then it was in essence bilateral symptomatic interval BC with compromised survival (Table 3, Figs. 2, 3). The 10-year OS 70.1% of Syn(N + Contra)PBC (Table 4, Fig. 3) was close to the 10-year OS 71–77% of SBBC reported in our previous study [8]. Hence the treatment of Syn(N + Contra)PBC should be intensified as symptomatic SBBC and the therapy of Meta(N + Contra)PBC should be similar as unilateral symptomatic interval BC. Syn(N + Contra)PBC and SynBiNPBC belonged to distinct clinical entities with different prognoses and thus should be treated differently (Table 4, Figs. 2, 3). Taken together, these results implied that the survival ordered from poor to favorable might be like: Syn(N + Contra)PBC ≤ (N + Contra)PBC ≤ Meta (N + Contra) PBC ≌ symptomatic unilateral BC (UBC) < MetaBiNPBC ≤ SynBiNPBC ≌ BiNPBC ≌ UniNPBC.

The key strength of this study was that the clinicopathological features and survival outcomes were compared between both UniNPBC vs BBC and among subgroups of BBC with long follow-up time of 48–227 (median 91) months. There are several limitations in our study. Firstly, although majority of BBC could not fully be explained by BRCA carriership [18, 21], there was limited germline mutation data about BRCA1/2 and other BC related genes in the current study. Several studies suggest that BBC is one of the related clinical factors that increases the probability of BRCA mutations [9, 22] and remains one of the criteria for recommendation of genetic testing [23]. BRCA mutation rate was about 24% among Chinese women with BBC [22]. Interval breast cancers among BRCA mutation carriers have worse clinicopathologic features than screen-detected tumors, and require more aggressive medical and surgical therapy [6]. Association between BRCA mutation and survival of BBC and screen-detected BBC would be further studied in our future research. Secondly, due to the BC screening conditions in China [3, 7, 12, 14, 15], there was no clear-cut documentation of whether the contralateral palpable BC was screen-detected symptomatic or interval BC between regular screenings, which had overlap but were not identical clinical entities [24]. So they were both regarded as ContraPBC in this study. Thirdly, it was a retrospective single-center study based on hospital population with limited case number. Thus comparison between NPBC as first cancer and NPBC as second cancer among Meta(N + Contra)PBC could not be performed. Last but not the least, the patients enrolled in this study were collected over 15 years (2013.01–2017.12), although the follow-up time was long enough to identify the metachronous BBC with long interval, the improvement of neo/adjuvant therapy would inevitably bring bias to prognosis of BBC due to the heterogeneity in BC treatment over decades.

## Conclusion

Compared to UniNPBC, patients with screen-detected bilateral BC had more invasive, ER negative, PR negative, triple-negative BC as well as less breast conserving surgery, radiotherapy, and endocrine therapy. Screen-detected bilateral NPBC including SynBiNPBC and MetaBiNPBC showed good prognosis as UniNPBC so that the therapy of BiNPBC could be de-escalated and optimized according to UniNPBC. Contrarily, screen-detected ipsilateral NPBC with contralateral palpable BC [(N + Contra) PBC] manifested unfavorable survival worse than UniNPBC (*p* < 0.001) and synchronous (N + Contra) PBC had the worst survival among all subgroups (*p* < 0.001), implying that these were actually bilateral interval BC and required intensified treatment.

## Supplementary Information

Below is the link to the electronic supplementary material.

Supplementary file1 (DOCX 146 kb)

## Data Availability

The datasets analyzed during the current study are available from the corresponding author on reasonable request.
