# Supplementary material for: Long-term survival of screen-detected synchronous and metachronous bilateral non-palpable breast cancer among Chinese women: a hospital-based study (2003–2017)
Source: Breast Cancer Res Treat. 2022 Sep 27;196(2):409–22. doi: 10.1007/s10549-022-06747-5 (PMC9581860; doi:10.1007/s10549-022-06747-5)
Supplement: Supplementary file 1 — Supplementary file1 (DOCX 146 kb) [file 10549_2022_6747_MOESM1_ESM.docx]

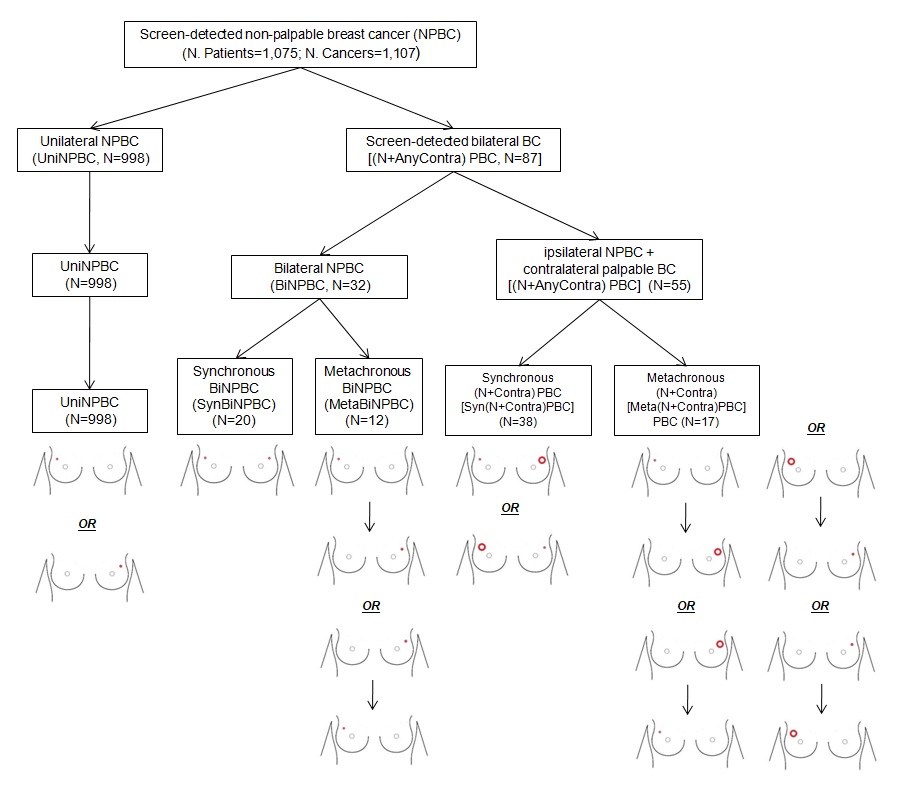


**Supplementary Figure**. Diagram of the subgroups of screen-detected bilateral breast cancer. The small red circle indicates the screen-detected NPBC, while the big red circle represents the palpable interval cancer. The black arrow indicates the detection sequence of tumors.
